# High-Emissivity M^2+^@TiO_2_/High-Entropy Coating on Flexible Aluminosilicate Fiber Fabric for Enhanced Bonding Strength and Thermal Insulation Performance

**DOI:** 10.3390/ma19112317

**Published:** 2026-05-31

**Authors:** Jiahui Xu, Xueying Zhang, Xiaohui Ma, Jiachen Liu

**Affiliations:** Key Laboratory of Advanced Ceramics and Machining Technology of Ministry of Education, School of Materials Science and Engineering, Tianjin University, Tianjin 300072, China; xujiahui_00@tju.edu.cn (J.X.); xiaohuima@tju.edu.cn (X.M.)

**Keywords:** high-emissivity coating, aluminosilicate fiber fabric, high-entropy titanate, bonding strength, interlayer

## Abstract

High-emissivity coatings on flexible fibrous fabrics are promising thermal-insulation materials for thermal protection systems in hypersonic vehicles. However, the interfacial bonding strength between the high-emissivity coating and the flexible fibrous substrate remains a critical challenge. A high-emissivity double-layer TiO_2_ interlayer modified by metal ions (Mg^2+^, Co^2+^, Ni^2+^, and Zn^2+^) with high-entropy (M^2+^@TiO_2_-HE) coating was developed on an aluminosilicate fiber fabric. The interlayer of the M^2+^@TiO_2_-HE coating not only enhances bonding strength through mechanical interlocking but also improves thermal insulation performance by acting as an infrared reflective layer. The bonding strength between the M^2+^@TiO_2_-HE coating and the ASFF substrate showed an 183% enhancement compared to that of the single-layer high-emissivity high-entropy coating. Under one-sided heating at 1400 °C, the backside temperature of the coated ASFF stabilizes at 330 °C, which is approximately 50 °C lower than that of the sample with the single-layer high-emissivity high-entropy coating. In the wavelength range of 0.3–2.0 μm, the average reflectivity of the M^2+^@TiO_2_ coating was 0.70, and in the wavelength range of 1–14 μm, the average emissivity of the M^2+^@TiO_2_-HE coating was 0.89. The M^2+^@TiO_2_-HE coating with high emissivity and reflectivity enhances both the bonding strength and thermal insulation performance of ASFF, showing its potential for applications in aerospace thermal protection systems.

## 1. Introduction

High-speed aerospace vehicles experience severe aerodynamic heating during service [1,2,3]; thus, reliable thermal protection systems (TPS) must be developed [4,5,6]. Flexible felt insulation, benefiting from its low weight, low thermal conductivity and enabling large-area forming, has become a mainstream material for TPS. Aluminosilicate fiber fabrics (ASFF) are frequently employed in flexible felt insulation owing to their good thermal stability, low thermal conductivity, and low coefficient of thermal expansion. However, the infrared emissivity and thermal insulation performance of ASFF remain insufficient [7,8,9,10,11]. High-emissivity coatings can effectively improve the emissivity of ASFF [12,13,14], but high infrared emissivity and strong interfacial bonding strength with the fibrous substrate are required.

Ideal coatings for TPS should have high emissivity, robust interfacial bonding, and low thermal conductivity. Selected emissive agents traditionally vary depending on the coating system, commonly including MoSi_2_ [15], SiC [16], SiO_2_ [17], SiB_2_ [18], and TaSi_2_ [19]. These conventional single- or binary-phase materials have performance limits; thus, high-entropy ceramics have attracted increasing attention in the field of thermal-insulation coatings due to their expanded compositional design space and entropy-stabilization effects [20,21,22,23,24]. Moreover, the severe lattice distortion and complex electronic structures inherent in high-entropy systems can significantly enhance phonon scattering and broaden infrared absorption bands, thereby enhancing emissivity [25,26]. Building upon these theoretical advantages, recent studies have developed several high-entropy high-emissivity coating systems [27,28,29,30,31]. Various compositions, such as (CrCoFeMnNi)_3_O_4_ spinels [32], multi-rare-earth hexaaluminates [33], and (YHoErYb)_2_SiO_5_, have demonstrated exceptional infrared emissivity, effectively overcoming the limitations of conventional oxides. Notably, high-entropy pseudobrookite titanates exhibit high infrared emissivity and good thermal insulation performance, making them promising for high-emissivity coating applications [34]. Despite these significant advancements in emissive performance, most existing high-entropy coatings are applied to rigid ceramic substrates or alloy surfaces, but their potential for use in flexible substrates remains largely underexplored. High-entropy ceramics can be incorporated into coating slurries as particulate emissive agents for flexible fibrous substrates. However, directly applying such single-layer coatings on flexible ceramic fiber fabric substrates may lead to limited interfacial bonding, thereby affecting coating integrity and long-term service reliability.

To improve the bonding strength between high-emissivity coatings and fibrous substrates, several structural optimization strategies have been explored, such as interfacial design [35], graded structures [36,37,38], and whisker reinforcement [39], among which interfacial design has emerged as a particularly effective approach. Among different interfacial design schemes, introducing a TiO_2_ reflective interlayer is especially promising, as it can not only provide a structural transition but also reflect thermal radiation propagating inward from the high-emissivity coating [40,41]. However, the design of TiO_2_ interlayers still requires further optimization to simultaneously improve interfacial bonding strength and compatibility with high-emissivity high-entropy titanate ceramic coatings, because a mismatch in thermal expansion coefficient may lead to interfacial detachment during drying or thermal exposure [42]. Metal ions doping can effectively regulate the composition and structure of TiO_2_ interlayers, thereby mitigating thermal expansion coefficient mismatch and alleviating interfacial thermal stress [43,44,45].

In this study, a TiO_2_ interlayer doped with four metal ions (M^2+^@TiO_2_), namely Mg^2+^, Co^2+^, Ni^2+^, and Zn^2+^, was prepared via a sol–gel route [46]. Subsequently, a high-emissivity topcoat was fabricated using high-entropy (Mg, Co, Ni, Zn)Ti_2_O_5_, as the emissive phase, thereby constructing a high-emissivity M^2+^@TiO_2_/high-entropy (M^2+^@TiO_2_-HE) coating on flexible fiber fabrics. The M^2+^@TiO_2_ interlayer acted as an effective transition layer between the flexible aluminosilicate fiber fabric and the high-emissivity high-entropy coating, thereby enhancing the interfacial bonding strength and contributing to improved mechanical strength and thermal insulation performance [47,48]. The formation and structural characteristics of the M^2+^@TiO_2_ interlayer were systematically analyzed. Furthermore, the interfacial bonding strength, emissivity, and thermal insulation performance of the samples subjected to different temperature treatments were investigated.

## 2. Materials and Methods

### 2.1. Materials

Aluminosilicate fiber fabric with a plain-weave structure was purchased from a domestic supplier. The fabric is composed of low-crystallinity alumina and amorphous silica, with a thickness of 0.40 ± 0.01 mm. Silica sol (particle size: 10 nm, solid content: 25%, pH = 7) was supplied by Jiangtian Chemical Co., Ltd. (Tianjin, China). The oxide powders used to prepare the high-entropy, high-emissivity coating fillers, including ZnO (99.9%), CoO (99.0%), MgO (99.9%), NiO (99.5%), and TiO_2_ (99.8%), were purchased from Shanghai Aladdin Biochemical Technology Co., Ltd. (Shanghai, China). Citric acid (CA, 99.9%) and tetrabutyl orthotitanate (TBOT, 99.7%) were purchased from Shanghai Macklin Biochemical Co., Ltd. (Shanghai, China). The metal nitrate hexahydrates used for preparing the M^2+^@TiO_2_ coatings, including Ni(NO_3_)_2_·6H_2_O (99.0%), Co(NO_3_)_2_·6H_2_O (99.0%), Mg(NO_3_)_2_·6H_2_O (99.6%), and Zn(NO_3_)_2_·6H_2_O (99.8%), were purchased from Tianjin Kaimate Chemical Technology Co., Ltd. (Tianjin, China). All chemicals and raw materials were used as received.

### 2.2. Preparation of the M^2+^@TiO_2_ Sol

**Figure 1:**
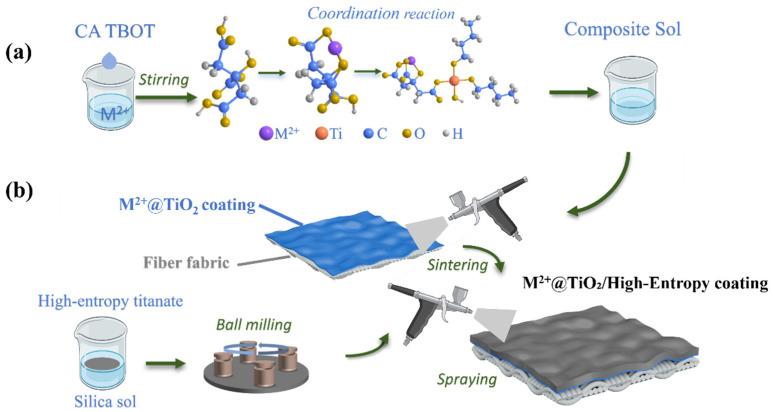
Schematic of the fabrication process for the double-layer coating on ASFF: (**a**) preparation of the M^2+^@TiO_2_ sol; (**b**) preparation of the M^2+^@TiO_2_-HE coating.

An M^2+^@TiO_2_ sol was prepared using CA, TBOT, and metal nitrate hexahydrates (Ni(NO_3_)_2_·6H_2_O, Co(NO_3_)_2_·6H_2_O, Mg(NO_3_)_2_·6H_2_O, and Zn(NO_3_)_2_·6H_2_O) as starting materials. CA and each metal nitrate hexahydrate were separately dissolved in ethanol and then mixed at prescribed ratios to obtain a mixture with an appropriate M^2+^/CA molar ratio. Subsequently, TBOT diluted with ethanol at a volume ratio of 1:1 was added dropwise into the M^2+^/CA mixture at a fixed M^2+^/TBOT molar ratio of 1:2. After further stirring for 1 h, a stable and homogeneous M^2+^@TiO_2_ sol was obtained, which transformed into a gel upon aging. The detailed preparation procedure is shown in Figure 1a.

### 2.3. Preparation of the M^2+^@TiO_2_-HE Coating

The M^2+^@TiO_2_ sol was uniformly sprayed onto the ASFF surface using a spray gun. The spraying pressure was set to 0.5 MPa, the effective spraying amount was controlled at 30–50%, and the areal density of the sprayed coating was maintained at 20 g/m^2^. The coating was applied in two passes to obtain a uniform and continuous layer. The samples were then allowed to dry naturally at room temperature for 12 h and subsequently heat-treated in a muffle furnace at various temperatures to form the M^2+^@TiO_2_ interlayer.

The high-entropy titanate ceramic powder was prepared through solid-state sintering. Five metal oxide powders were weighed according to their stoichiometric ratios and ball-milled with ethanol as the dispersion medium. Ball-milling was performed at 200 rpm for 6 h, with both the powder-to-ethanol and ball-to-powder mass ratios set to 1:1. The resulting slurry was dried in an oven at 60 °C for 12 h and then ground and passed through a 60-mesh sieve to obtain a homogeneously mixed precursor powder. The powder was then sintered at 1400 °C to obtain the high-entropy titanate ceramic, which was subsequently ball-milled again for 6 h to yield the final high-entropy titanate ceramic powder.

A high-emissivity coating slurry was prepared using the high-entropy titanate ceramic powder as the filler and silica sol as the binder. The slurry was then sprayed onto the surface of the above-mentioned M^2+^@TiO_2_ interlayer. After natural drying for 12 h, the M^2+^@TiO_2_-HE coating was obtained. The specific preparation process is shown in Figure 1b.

### 2.4. Characterization

**Figure 2:**
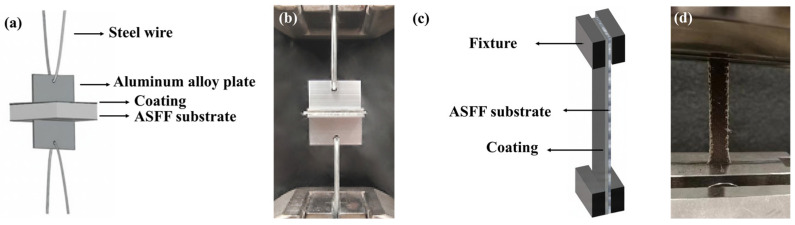
(**a**) Schematic diagram of bonding strength test; (**b**) photograph of bonding strength test; (**c**) schematic diagram of tensile strength test; (**d**) photograph of tensile strength test.

A sedimentation experiment was conducted to evaluate the dispersion stability of the unmodified TiO_2_ sol and the M^2+^@TiO_2_ sol. Fourier-transform infrared (FTIR) spectra of the M^2+^@TiO_2_ sol and the ASFF with M^2+^@TiO_2_ coating were recorded using an FTIR spectrometer equipped with an attenuated total reflectance (ATR) accessory (INVENIO S, Bruker, Ettlingen, Germany). The spectral range was 4000–500 cm^−1^, the resolution was 4 cm^−1^, and 64 scans were accumulated to improve the signal-to-noise ratio. The phase compositions of the M^2+^@TiO_2_ sol and the ASFF with corresponding M^2+^@TiO_2_ coatings before and after heat treatment were analyzed via X-ray diffraction (XRD, D/MAX-2500, Rigaku, Akishima, Japan) over a 2θ range of 10–90°, and the surface morphologies of the coatings were observed via scanning electron microscopy (SEM, ZEISS SIGMA 300, Oberkochen, Germany). The tensile strength of the coated ASFF samples was measured using a universal testing machine (CMT4303, Meister Industrial System, Shanghai, China), and the same machine was used to measure the bonding strength between the coating and the ASFF according to GB/T 5210-2006 [49]. In the bonding-strength test, two aluminum plates (30 mm × 30 mm) were used as auxiliary loading fixtures, and high-strength double-sided tape was employed only for specimen mounting and load transfer. The coated ASFF sample (30 mm × 30 mm × 0.4 mm) was sandwiched between the two aluminum plates, and axial tension was applied at a rate of 0.5 mm min until the coating was completely peeled from the ASFF substrate. The maximum bonding strength was calculated from the peak force recorded during the test and the effective bonded area. As the coating covered the full surface of the specimen, the effective bonded area was considered to be 30 mm × 30 mm. The coating was evaluated using the test setups shown in Figure 2. Five specimens were tested for each group, and the average value was reported.

For the thermal-protection test, the coating was applied to one side of the ASFF, and a 10 mm-thick quartz fiber blanket was mechanically attached to the opposite side. The heating condition was first calibrated using an uncoated ASFF specimen as a reference. Specifically, the flame intensity of the butane torch and the distance between the torch and the specimen were adjusted until the front-surface temperature of the uncoated ASFF reached 1400 °C. Once this condition was established, both the flame intensity and the torch-to-sample distance were fixed and used for all coated samples. The coated side of each sample was then subjected to single-sided flame heating, and the temperature evolution on both the front and back sides was monitored using a thermal infrared imaging system (LT7-P, DALI Technology Co., Ltd., Hangzhou, China).

## 3. Results

### 3.1. Characterization of M^2+^@TiO_2_ Coatings

**Figure 3:**
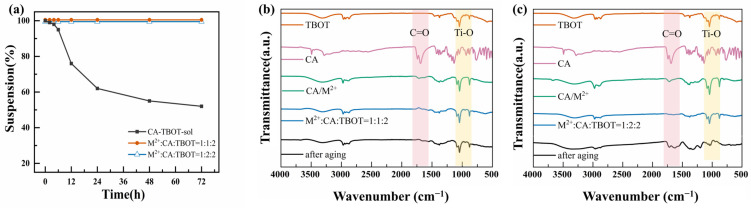
Stability and chemical characterization of M^2+^@TiO_2_ sols: (**a**) sedimentation stability of M^2+^@TiO_2_-1 and M^2+^@TiO_2_-2 sols; FTIR spectra of precursor solutions and aged M^2+^@TiO_2_-1sol (**b**) and M^2+^@TiO_2_-2sol (**c**).

The stability of the TiO_2_ sol and the M^2+^@TiO_2_ sol was evaluated using a sedimentation method, and the suspension ratio was calculated as the ratio of the sedimented layer height to the initial height of the suspension in the container. Figure 3a shows that the TiO_2_ sol without metal ions readily underwent hydrolysis in air and formed precipitated TiO_2_ particles. Its suspension ratio decreased to 76% and 52% after 12 and 72 h of standing, respectively. The formation of agglomerates and sedimentation would severely compromise the subsequent spray-coating process. In contrast, the M^2+^@TiO_2_ sol exhibited markedly improved stability and remained clear even after exposure to air for 72 h. This enhanced stability is primarily attributed to the synergistic effects of multiple metal ions, which effectively reduce the reactivity of free titanate species, thereby suppressing rapid hydrolysis and polycondensation in the presence of trace water. Figure 1a shows that Ti ions participate in coordination reactions to form multinuclear complexes with dual coordination environments [50,51], effectively stabilizing the titanium species in the sol and preventing the rapid hydrolysis/condensation of TBOT that would otherwise yield TiO_2_ precipitates. The excellent stability of the M^2+^@TiO_2_ sol ensures the feasibility of preparing the M^2+^@TiO_2_ coating and favors the formation of a more uniform and intact coating.

To further investigate the coordination reactions among CA, metal ions, and TBOT, FTIR spectra were collected for TBOT, CA, mixtures of CA and metal ions, and the sol systems before and after aging. The molar ratios of M^2+^/CA/TBOT were set to 1:1:2 and 1:2:2, respectively. The resulting sols were denoted as M^2+^@TiO_2_-1 (Figure 3b) and M^2+^@TiO_2_-2 (Figure 3c), respectively. After citric acid was mixed with the ethanol solution of metal ions, the characteristic carboxyl absorption band of citric acid near 1700 cm^−1^ was significantly weakened, indicating that coordination had occurred [52]. In the FTIR spectrum of TBOT, the Ti-O framework vibration band is located in the range of 700–900 cm^−1^, while a strong Ti-O-C stretching band appears near 1100 cm^−1^ [53]. The shift in this band suggests that Ti species participate in coordination after hydrolysis and interact with the carboxyl groups of citric acid, causing electron-withdrawing groups to influence the Ti-O framework vibrations. In the M^2+^@TiO_2_-2 sol system, citric acid not only chelates Ti species independently but is also more likely to form heterometallic complexes that bridge M^2+^ and Ti. Compared with the M^2+^@TiO_2_-1 sol, this structure introduces greater steric hindrance and more effectively suppresses the hydrolysis and polycondensation of TBOT [54,55,56]. Consequently, its sol stability is superior to that of the pristine titanium system and the M^2+^@TiO_2_-1 sol.

**Figure 4:**
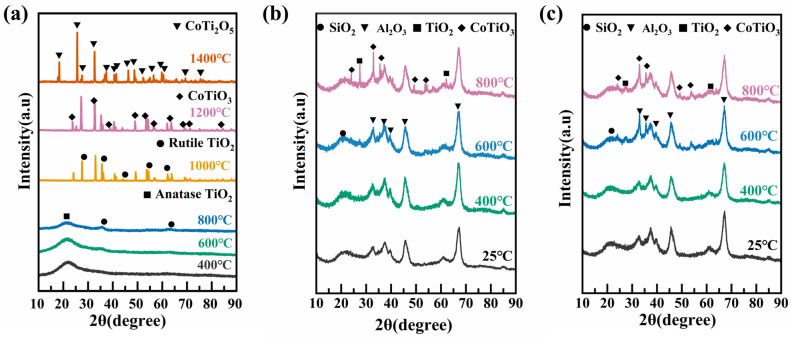
XRD analysis of phase evolution in the M^2+^@TiO_2_ system: (**a**) xerogel was obtained from the M^2+^@TiO_2_-2 sol via aging and drying, and then heat treatment at different temperatures; (**b**) M^2+^@TiO_2_-1 and (**c**) M^2+^@TiO_2_-2 coatings sprayed on the ASFF surface and heat-treated at different temperatures.

Figure 4a shows the XRD patterns of the dried gels after heat treatment at different temperatures. The results indicate that the dried gel remained largely amorphous below 800 °C, and crystalline phases, mainly TiO_2_, gradually precipitated with increasing temperature. Upon further heating, TiO_2_ underwent phase transformation and, together with multiple cations, formed perovskite-type titanate phases through solid-solution reactions. When the temperature was increased to 1400 °C, the residual TiO_2_ and perovskite-type titanates reacted to form a high-entropy pseudobrookite-type titanate phase. Variable-temperature XRD analysis of the aluminosilicate fiber fabrics coated with the two-sol formulations is shown in Figure 4b,c. At 25 °C and 400 °C, the diffraction signals are dominated by the Al_2_O_3_ crystalline peaks of the substrate fibers and the broad diffuse halo of SiO_2_, with no obvious new crystalline phase observed, indicating that the coating precursor remains in an amorphous or poorly ordered state. When the temperature reaches 600 °C, structural rearrangement and crystallization of the coating begin. At 800 °C, characteristic diffraction peaks corresponding to TiO_2_ and CoTiO_3_ appear, indicating that the sol undergoes in situ ceramicization on the fiber surface and transforms into titanium oxide and titanate phases. Although the two coatings (M^2+^@TiO_2_-1 in Figure 4b and M^2+^@TiO_2_-2 in Figure 4c) follow essentially the same phase evolution path from 25 to 800 °C, the formulation with a higher CA content (Figure 4c) exhibits more pronounced and sharper TiO_2_ and ilmenite-type structure diffraction peaks in the 600–800 °C range, suggesting more homogeneous coordination of the precursor species and a more uniform distribution of metallic species in the sol stage.

**Figure 5:**
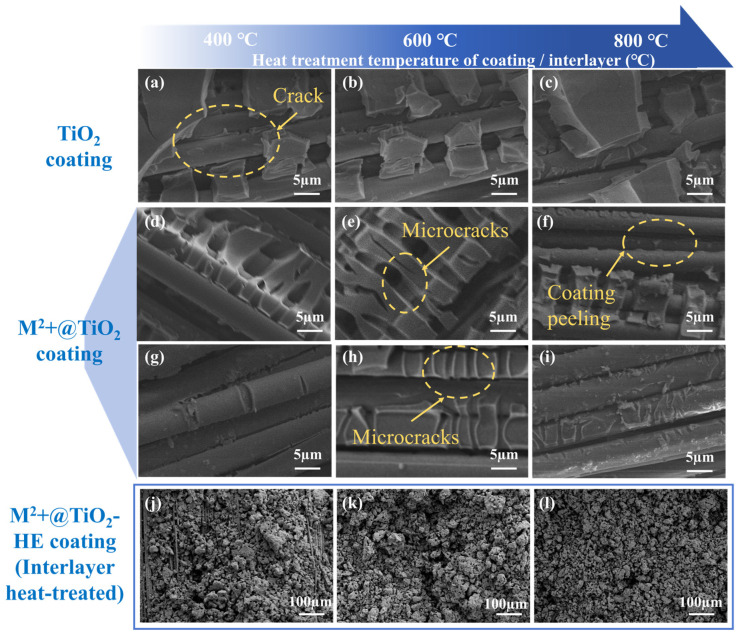
Surface morphologies of the M^2+^@TiO_2_ interlayer and the M^2+^@TiO_2_-HE coating: (**a**–**c**) SEM images of the TiO_2_, (**d**–**f**) M^2+^@TiO_2_-1, (**g**–**i**) M^2+^@TiO_2_-1 and (**j**–**l**) M^2+^@TiO_2_-HE coatings.

Figure 5a–i compare the morphological evolution of the TiO_2_ coating and the M^2+^@TiO_2_ composite coating after heat treatment at 400, 600, and 800 °C. For the pure TiO_2_ coating, obvious cracks already appear at 400 °C, indicating early damage caused by shrinkage and thermal stress during calcination. With increasing temperature, the coating becomes further fragmented and increasingly discontinuous. In contrast, the M^2+^@TiO_2_ coating retains a better overall integrity and develops uniformly distributed microcracks at 600 °C. Crucially, these microcracks can provide mechanical interlocking between the subsequent high-entropy coating and the aluminosilicate fiber fabric, thereby enhancing the interfacial bonding strength. At 800 °C, the M^2+^@TiO_2_-1 coating exhibits local peeling, whereas the M^2+^@TiO_2_-2 coating remains intact throughout. Therefore, the M^2+^@TiO_2_-2 coating formulation treated at 600 °C, which exhibits the most uniform microcracks and high structural integrity, was selected as the optimal intermediate layer.

### 3.2. Performance of the M^2+^@TiO_2_-HE Coating

Based on the aforementioned results, the sol formulation with optimal ratio and sintering temperature was selected to prepare the optimal interlayer. At this temperature, the coating maintains superior structural integrity and a uniform distribution of microcracks (approximately), which facilitates microcrack toughening. Subsequently, a high-emissivity layer based on high-entropy titanate was deposited onto this optimized interlayer, yielding the microstructures shown in Figure 5j–l. The high-entropy titanate particles are uniformly distributed across the modified fiber surface, exhibiting robust interfacial bonding with the underlying layer. The comprehensive performance of the resulting double-layer HE coating was then systematically investigated.

**Figure 6:**
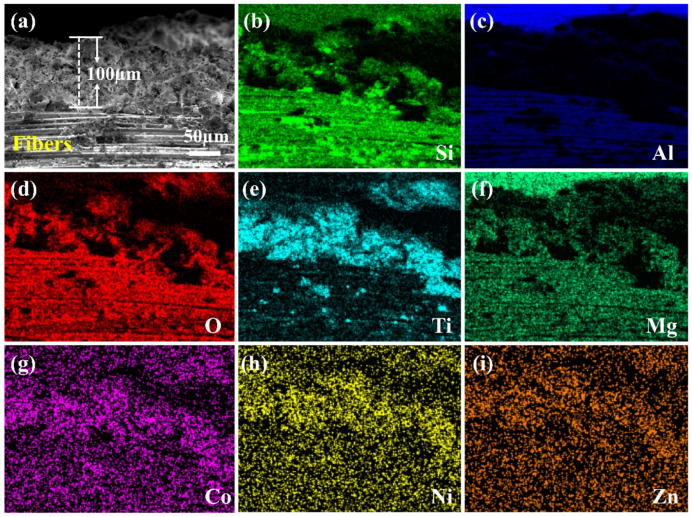
EDS area scan analysis of the cross-section of fiber fabric with M^2+^@TiO_2_-HE coating (**a**) SEM; (**b**) Si, (**c**) Al, (**d**) O, (**e**) Ti, (**f**) Mg, (**g**) Co, (**h**) Ni, and (**i**) Zn.

Figure 6 presents the cross-sectional elemental mapping of the fiber fabric coated with the M@TiO_2_-HE interlayer. The cross-section of the double-layer coating is continuous and relatively dense, with an overall thickness of approximately 100 μm. The interlayer uniformly covers the fiber surface. Figure 6e–h show that Ti, Mg, Co, Ni, and Zn are evenly distributed within the high-emissivity layer, confirming that the titanate phase used as the emissive component indeed forms a stable and homogeneous high-entropy phase. The Si and O signals in the emissive layer mainly originate from the silica sol used as a high-temperature binder.

**Figure 7:**
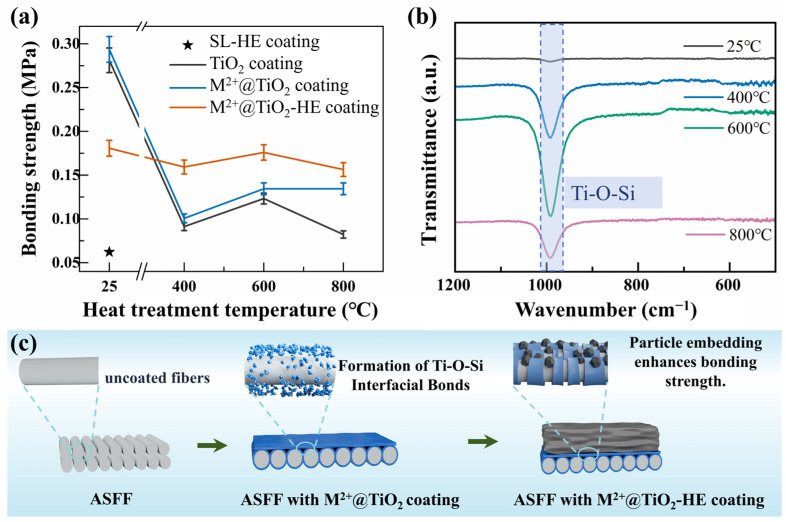
Interfacial bonding and adhesion properties of the coatings on ASFF: (**a**) bonding strength of different coatings after heat treatment at various temperatures; (**b**) FTIR spectra of the ASFF with the M^2+^@TiO_2_ coating; (**c**) dual reinforcement mechanism of the surface bonding strength for the ASFF with the M^2+^@TiO_2_-HE coating.

The interfacial bonding strength of the samples was measured using a universal testing machine to evaluate the influence of the M^2+^@TiO_2_ coating on the interface performance of the aluminosilicate fiber fabric. As shown in Figure 7a, the interfacial bonding strength is strongly affected by the heat-treatment temperature of the interlayer. The TiO_2_ and M^2+^@TiO_2_ layers were prepared at room temperature via a sol–gel method using citric acid, metal salts, ethanol, and tetrabutyl titanate as precursors, followed by heat treatment at different temperatures. For the M^2+^@TiO_2_-HE sample, the heat-treatment temperature specifically refers to that of the interlayer prior to room-temperature spraying. At 25 °C, the TiO_2_ and M^2+^@TiO_2_ coatings exhibit relatively high bonding strength, mainly due to the organic species in the interfacial layer that bridge the coating and the fiber substrate. At 400 °C, both the bonding strength and tensile strength decrease significantly, which is mainly associated with the extensive burnout of organic components in the range of 25–400 °C. When the temperature is increased to 600 °C, the performance of the M^2+^@TiO_2_ sample improves, which, according to the FTIR analysis in Figure 7b, can be attributed to the more sufficient formation of Ti-O-Si bonds [57,58,59,60] and the resulting enhancement of interfacial bonding. At 800 °C, the bonding strength decreased again, which was mainly attributed to the increased crystallinity of TiO_2_ in the coating at elevated temperature and the transformation of oxides into titanate phases (Figure 4). Meanwhile, high-temperature sintering led to further shrinkage and even partial delamination of the coating (Figure 5).

The improved adhesion associated with the microcracked structure can be understood from two aspects. First, the increased surface roughness enlarges the actual contact area and provides more anchoring sites for the coating, thereby enhancing mechanical interlocking. Second, such a structure helps relieve part of the stress and reduce the deformation mismatch between the substrate and the coating [61]. For the M^2+^@TiO_2_-HE coating, the Ti-O-Si bonds formed between the M^2+^@TiO_2_ interfacial layer and the fiber substrate strengthen the bonding between them. Meanwhile, after heat treatment at 600 °C, the interfacial layer develops a microcracked structure (Figure 5), which facilitates the embedment of particles of the top high-entropy coating and thereby improves the coating adhesion. Consequently, the M^2+^@TiO_2_-HE sample maintains relatively high bonding strength over the temperature range of 400–800 °C and reaches a higher value at 600 °C, with the variation in its bonding strength with temperature similar to that of the M^2+^@TiO_2_ coating.

**Figure 8:**
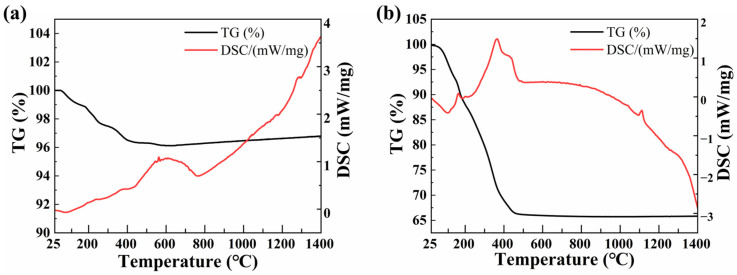
Thermal behavior of the M^2+^@TiO_2_-2 system: (**a**) xerogel derived from the M^2+^@TiO_2_ sol; (**b**) the ASFF with the M^2+^@TiO_2_ sol coating.

To investigate the thermal evolution of the coatings, TG-DSC analysis was performed on the TiO_2_ and M^2+^@TiO_2_ sol precursors (Figure 8). The results indicate that the system undergoes vigorous decomposition of organic components at temperatures up to 400 C. The resulting mass loss of approximately 24–35% leads to significant volumetric shrinkage, which triggers the formation of visible cracks on the coating surface. Further increases in temperature cause the TG curves to reach a plateau, suggesting the near-complete removal of organic constituents. This transition is accompanied by the further sintering and condensation of inorganic oxide components, which promotes the formation of a more robust transition layer on the fiber surface.

**Figure 9:**
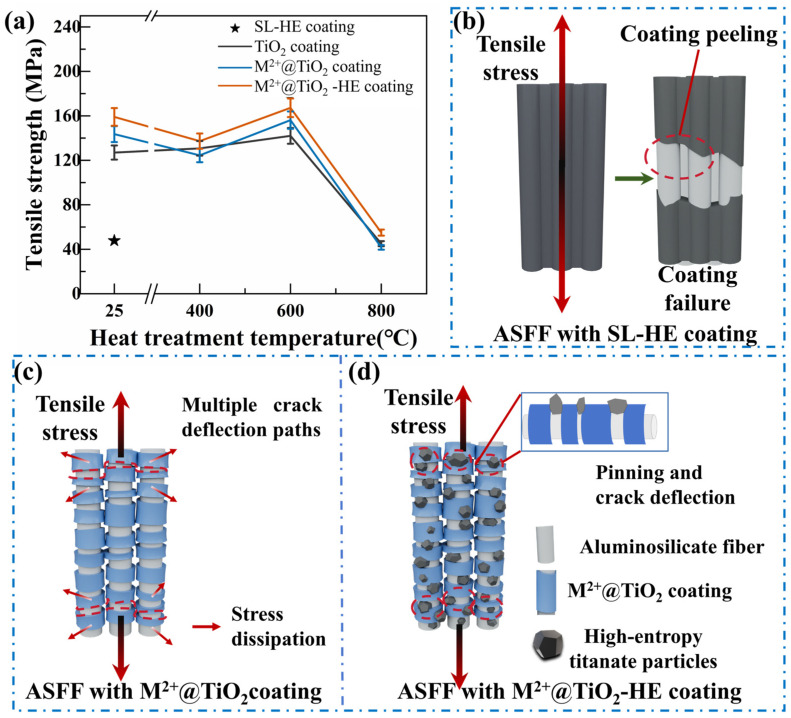
Mechanical performance and toughening mechanisms of the coated ASFF: (**a**) tensile strength of the ASFF with different coatings after heat treatment; (**b**–**d**) diagrams illustrating the stress distribution, crack deflection, and synergistic toughening effects of the M^2+^@TiO_2_-HE double-layer coating.

The macroscopic mechanical performance of the coated ASFF was evaluated by tensile testing over the temperature range of 25–800 °C, as shown in Figure 9a. The sample with the single-layer high-entropy coating exhibits relatively low tensile strength, indicating its poor resistance to tensile damage. In contrast, the TiO_2_, M^2+^@TiO_2_, and M^2+^@TiO_2_-HE coated samples all show significantly higher tensile strength in the temperature range of 25–600 °C, among which the M^2+^@TiO_2_-HE coating exhibits the best overall performance. In particular, the tensile strength of all coated samples reaches a relatively high level at 600 °C, but decreases markedly after heat treatment at 800 °C. This variation is mainly associated with the phase transformation of the coatings during heat treatment. As shown by the XRD results in Figure 4, when the temperature increases to 800 °C, the crystallinity of Al_2_O_3_ and SiO_2_ in the fiber substrate increases, which disrupts the internal fiber structure, while the amorphous oxides in the coating transform into titanate phases, possibly leading to increased brittleness of the coating and thereby reducing the tensile performance. Meanwhile, the surface morphologies shown in Figure 5 indicate that high-temperature sintering at 800 °C causes coating shrinkage, crack widening, and even local peeling, thereby weakening the overall integrity of the coating system and resulting in the deterioration of tensile performance.

The failure mechanisms shown in Figure 9b–d further explain the tensile behavior described above. For the single-layer high-entropy coating, its relatively dense structure makes it prone to large-area coating spallation under tensile loading, ultimately leading to failure. After introducing the M^2+^@TiO_2_ interfacial layer, the coating system exhibits improved tensile resistance, because the microcrack structure within the M2+@TiO2 interfacial layer can provide multiple crack-deflection paths, thereby dissipating part of the applied stress [61]. For the M^2+^@TiO_2_-HE coating, in addition to the enhancement in tensile resistance provided by the microcrack structure of the M2+@TiO2 interfacial layer, the high-entropy titanate particles in the top high-entropy coating can become embedded in the microcracks to form a pinning effect, which induces crack deflection and suppresses crack propagation, thereby maintaining the stability of the coating under tensile loading [62]. Therefore, the M^2+^@TiO_2_-HE coating exhibits excellent tensile resistance and shows great potential for application in high-emissivity coatings.

**Figure 10:**
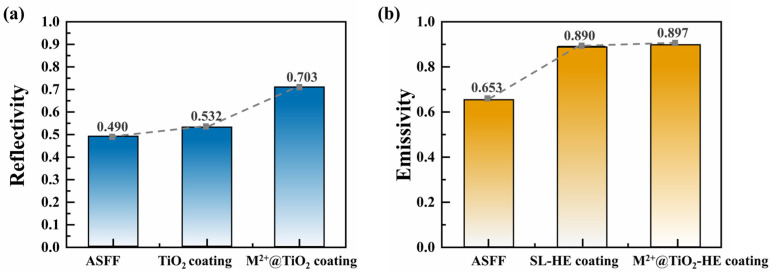
(**a**) Average reflectivity of ASFF substrates with different coating configurations in the 0.3–2 μm range. (**b**) Average emissivity of ASFF substrates with different coating configurations in the 1–14 μm range.

The superior thermal-protection behavior of the double-layer coating can be understood from the perspective of spectrally differentiated radiative heat transfer. Under one-sided high-temperature flame exposure, the incident thermal radiation contains a substantial short-wavelength component with high radiative intensity. In this spectral range, the M^2+^@TiO_2_ interlayer acts as an efficient reflective barrier, increasing the average reflectivity from 0.490 for bare ASFF and 0.532 for the TiO_2_-coated sample to 0.703 (Figure 10a), thereby reducing radiative energy absorption by the substrate. The high-entropy titanate top layer exhibits a high infrared emissivity (0.897), markedly higher than that of bare ASFF (0.653), which promotes re-radiation of the absorbed heat in the infrared range. Therefore, the functions of high reflectivity and high emissivity are not contradictory but complementary: the former suppresses the incoming radiative heat load, while the latter enhances outward thermal radiation from the heated surface. Through this wavelength-selective synergy, the double-layer coating reduces the net radiative heat transfer into the ASFF and thus improves the macroscopic thermal-insulation performance under one-sided high-temperature heating.

**Figure 11:**
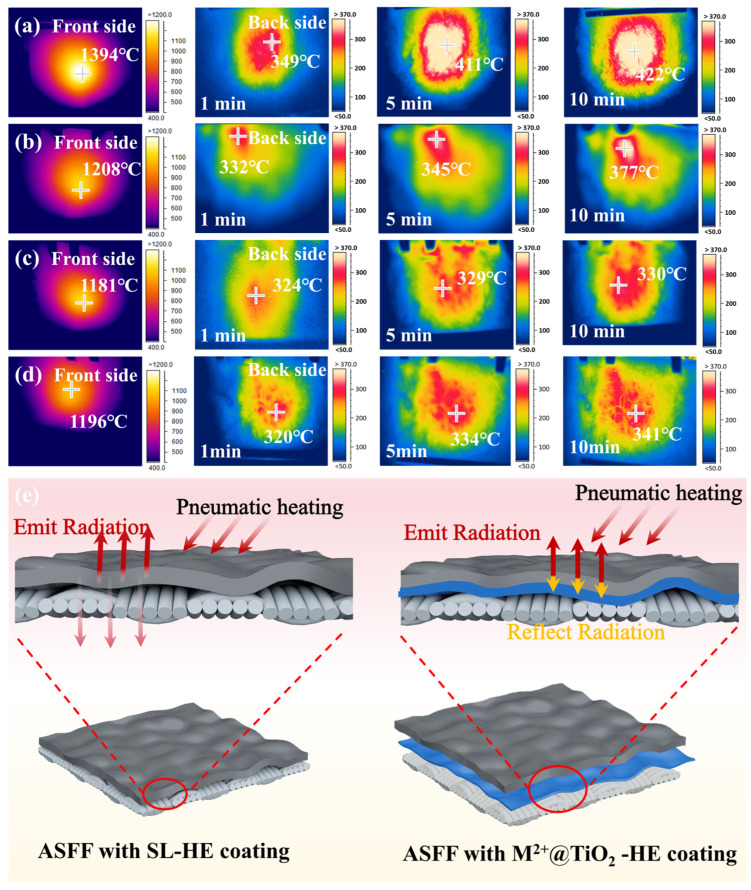
Thermal protection performance and infrared radiation mechanism of the M^2+^@TiO_2_-HE coating: (**a**–**c**) infrared thermal images of the front and back sides of ASFF with different coatings under one-sided flame heating; (**d**) infrared thermal images of the M^2+^@TiO_2_-HE-coated specimen subjected to five flame-heating cycles; (**e**) schematic of the radiation reflection and emission mechanisms of the M^2+^@TiO_2_-HE coating.

Figure 11 shows that the thermal protection performance of the fiber substrate is progressively improved by surface coating modification. The bare aluminosilicate fiber fabric in Figure 10a exhibits the weakest thermal-shielding capability because the flame directly impinges on the exposed fiber surface, allowing heat to penetrate rapidly into the interior. Consequently, its front surface temperature reaches as high as 1394 °C, and the backside temperature rises to 422 °C after 10 min of exposure. In comparison, the single-layer HE coating in Figure 11b provides a clear improvement, reducing the front and backside temperatures to 1208 °C and 377 °C, respectively. This indicates that the coating can partially reflect, scatter, and block the incident heat flux. However, the protection effect of the single-layer structure remains limited under prolonged flame exposure, likely because the heat-transfer path is still relatively direct and the coating is more susceptible to local thermal damage or structural degradation. By contrast, the M^2+^@TiO_2_-HE coating in Figure 11c shows the most effective thermal-barrier performance, maintaining the lowest front surface temperature of 1181 °C and a backside temperature of merely 330 °C under the same testing conditions. To further evaluate the thermal stability and cyclic reliability of the double-layer coating, the same M^2+^@TiO_2_-HE-coated specimen was subjected to five consecutive flame-heating cycles under identical conditions, and the corresponding infrared thermal images are presented in Figure 11d. After five heating cycles, the coated sample still maintained a relatively low front-surface temperature of 1196 °C, while the backside temperatures were 320 °C, 334 °C, and 341 °C at 1, 5, and 10 min, respectively. Compared with the first test shown in Figure 11c, only a slight increase in the front and back side temperatures was observed after repeated thermal exposure, indicating that the coating retained most of its thermal-protection capability. After 5 cyclic heating tests, the bonding strength was 0.17 MPa. The results demonstrate that the M^2+^@TiO_2_-HE coating architecture possesses good thermal stability and structural durability under cyclic flame heating.

This superior result can be attributed to the synergistic effects of the rationally designed double-layer architecture. Specifically, the top high-entropy coating features severe lattice distortion, which significantly enhances phonon scattering, reduces intrinsic thermal conductivity, and provides strong radiative shielding against the high-temperature flame. Concurrently, the M^2+^@TiO_2_ interlayer serves dual functions. On one hand, the TiO_2_ component acts as an effective reflective agent that backscatters incident thermal radiation, further reducing heat penetration and contributing to the significant drop in surface temperature. On the other hand, it acts as a crucial structural buffer; its tailored microcrack network effectively accommodates the severe thermal mismatch stress induced by rapid heating, preventing the top HE coating from peeling off and thereby preserving the continuity and structural integrity of the entire thermal barrier. Combined with the excellent interfacial bonding strength shown in Figure 7a and the improved mechanical toughness in Figure 9, the M^2+^@TiO_2_-HE coating is able to maintain an intact protective barrier under extreme thermal exposure. Therefore, the thermal-protection behavior observed in Figure 11 clearly demonstrates that the multilayer high-entropy design is highly effective in protecting ASFF against severe heat flux.

**Figure 12:**
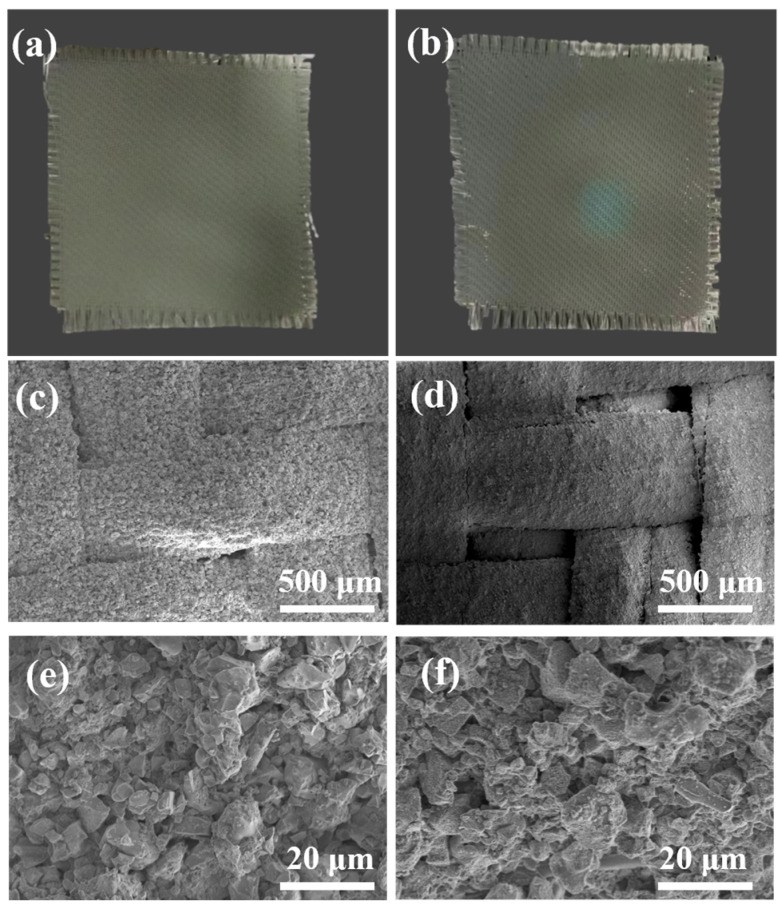
Comparison of the specimen morphology before and after the one-sided flame heating test: (**a**,**c**,**e**) macroscopic appearance, morphology, and SEM image before testing; (**b**,**d**,**f**) corresponding morphologies after the one-sided flame heating test.

Figure 12 shows that the sample largely retained its macroscopic integrity after the one-sided flame heating test, with only slight surface discoloration observed. This phenomenon may be attributed to the high-temperature melting of the silica sol and the further reaction of oxides in the interfacial layer to form the same high-entropy titanate phase as that in the top layer (Figure 4). Microscopic examination after flame exposure revealed no significant changes in the coating surface morphology, indicating that the coating largely preserved the integrity of the protective layer under flame exposure. This structural stability is considered beneficial for maintaining the thermal protection of the substrate during heating, and no obvious coating cracking or erosion was observed. These results suggest that the coating possesses relatively good high-temperature stability and interfacial adhesion, consistent with the thermal protection behavior discussed above.

**Figure 13:**
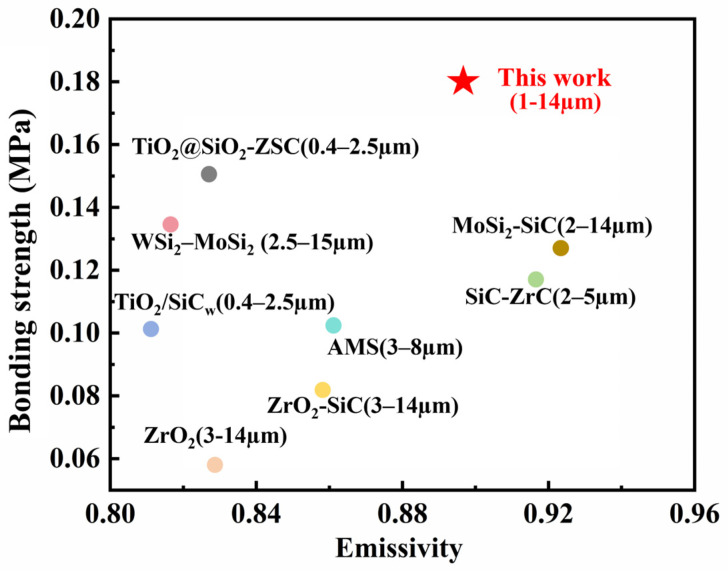
Bonding strength and emissivity of high-emissivity coatings in different works [12,13,14,18,35,42].

As shown in Figure 13, compared with previously reported high-emissivity coatings, the coating developed in this work combines high emissivity and bonding strength, demonstrating favorable overall performance together with excellent radiation performance.

## 4. Conclusions

A novel high-emissivity double-layer coating system comprising an M^2+^@TiO_2_ interlayer and a high-entropy top layer was successfully developed on an ASFF. The tailored M^2+^@TiO_2_ interlayer features a microcrack network that effectively accommodates thermal mismatch stress, significantly enhancing the mechanical stability. As a result, the tensile strength of the ASFF with the M^2+^@TiO_2_-HE coating reaches 167 MPa and the bonding strength reaches 0.1759 MPa. Furthermore, the double-layer system leverages a synergistic emission-reflection thermal shielding mechanism. The high-entropy top layer provides an exceptional infrared emissivity of up to 0.89 for efficient radiative cooling, while the M^2+^@TiO_2_ interlayer offers a high short-wavelength reflectivity of 0.70. Consequently, in high-temperature flame impingement tests, the ASFF with the M^2+^@TiO_2_-HE coating exhibits outstanding thermal insulation performance, reducing the front surface temperature from 1394 °C to 1181 °C and the backside temperature from 422 °C to 330 °C. The M^2+^@TiO_2_-HE coating shows promising potential for application in flexible aerospace thermal protection systems.

## Data Availability

The original contributions presented in this study are included in the article. Further inquiries can be directed to the corresponding authors.

## Abbreviations

The following abbreviations are used in this manuscript:

HE	High-entropy emissive topcoat based on pseudobrookite (Mg, Co, Ni, Zn)Ti_2_O_5_
M^2+^	Mg^2+^, Co^2+^, Ni^2+^, and Zn^2+^
M^2+^@TiO_2_ interlayer	M^2+^-modified TiO_2_ interlayer
M^2+^@TiO_2_-HE coating	Double-layer coating system composed of an M^2+^@TiO_2_ interlayer and an HE topcoat
ASFF	Aluminosilicate flexible fiber felt
